# Improvement of the Outcome of Relapsed or Refractory Acute Lymphoblastic Leukemia in Children Using a Risk-Based Treatment Strategy

**DOI:** 10.1371/journal.pone.0160310

**Published:** 2016-09-15

**Authors:** Francesco Ceppi, Michel Duval, Jean-Marie Leclerc, Caroline Laverdiere, Yves-Line Delva, Sonia Cellot, Pierre Teira, Henrique Bittencourt

**Affiliations:** Hemato-Oncology Division, Charles Bruneau Cancer Research Center, CHU Ste-Justine, Department of Pediatrics, University of Montreal, Montreal, Quebec, Canada; Queen's University Belfast, UNITED KINGDOM

## Abstract

Relapsed/refractory acute lymphoblastic leukemia (ALL) is a leading cause of death by cancer in children. Our institution has switched relapse treatment strategy to improve survival. We reviewed records of first relapse/refractory childhood ALL between 1996 and 2012. Based on length of first remission, relapse site and immunophenotype, patients were classified into two groups: standard-risk relapse (SRR) and high-risk relapse and refractory (HRRR). Before 2007, all patients were uniformly treated with the same induction as at presentation, followed by hematopoietic stem cell transplantation (HSCT). Since 2007, treatment was given according to risk of failure: SRR were mostly treated with chemotherapy; HRRR patients underwent HSCT after intensive chemotherapy, aiming reduction of pre-transplant disease burden. Sixty-four patients were included. Thirty (47%) were SRR and 34 (53%) HRRR, including 11 with refractory ALL. Five-years overall survival (OS) and event-free survival (EFS) were similar for SRR, but were significantly higher with new risk-based strategy for HRRR: 56% versus 17% (P = 0.03) for OS, and 56% vs 11% for EFS (P = 0.008), respectively. In multivariate analysis, treatment strategy was significantly associated with survival. In conclusion, change for a risk-based strategy in our institution increased survival of high-risk patients to levels similar of those of standard-risk patients.

## Introduction

Acute lymphoblastic leukemia (ALL) is the most common pediatric malignancy. Long-term survival has become the reality for the majority of children diagnosed with ALL, with approximately 85% [1, 2] surviving 5 years or longer after diagnosis. However, depending on certain risk factors, such as age at diagnosis, white blood cell (WBC) count at presentation, hematopoietic lineage, and cytogenetic abnormalities, approximately 15% will experience relapse, making recurrent ALL a relatively common situation in pediatric hemato-oncology [3]. Second remission rates range from 71% to 93%, and long-term survival rates from 40% to 50%, making recurrent ALL one of the leading cause of death by cancer in children [4–7]. Survival rates of relapsed patients depend on the site of relapse (isolated extramedullary disease showing the best response to treatment) and length of first complete remission (those relapsing during the treatment or shortly after stopping chemotherapy having the worst outcome).

Although primary induction failure is rare at presentation, occurring in only 2 to 3% of patients, refractory ALL also remains a therapeutic challenge. Indeed, a meta-analysis reported 10-year survival rate to be 32% for 1041 patients treated by 14 cooperative study groups between 1985 and 2000 [8].

The challenge thus is to devise strategies for the treatment of relapsed and refractory disease. Reinduction of relapsed ALL commonly includes conventional agents similar to those used at initial diagnosis. Hematopoietic stem cell transplantation (HSCT) is often used as consolidation therapy. Early minimal residual disease (MRD) response is a strong predictor of outcome [9]. The absence of detectable MRD at the end of reinduction therapy is associated with a better outcome in early-relapse as well as in late-relapse patients [5].

We modified our strategy for relapsed patients in 2007, in an attempt to increase survival. Before 2007, all relapsing patients were treated the same way, irrespective of their risk of treatment failure. They received the same induction as at first diagnosis. All patients in the second remission after the induction proceeded immediately to HSCT. This strategy led to overall survival (OS) of 40%. The outcome of patients with early marrow relapse was particularly dismal, as their 5-year-estimate OS was only 13% [10]. In 2007, we decided to base our strategy upon the risk of treatment failure. We thus introduced two major modifications in order to improve the outcome of patients with high-risk relapse or refractory (HRRR) disease without compromising the outcome of other patients. In an attempt to increase survival of higher risk patients, we intensified reinduction chemotherapy to decrease as much as possible residual leukemia before HSCT [11]. In an attempt to reduce toxicity in lower risk patients, HSCT was offered only to patients with early marrow relapse or late marrow relapse (the latter if they had a HLA-identical related donor or if an adverse cytogenetic features was present, such as t(9;22) or MLL rearrangement). Patients with extramedullary relapse and patients with late marrow relapse but without an HLA-identical related donor were treated with chemotherapy alone, following risk-based protocols.

The aim of this study was to compare the outcomes of these two different strategies. Our primary hypothesis was that event-free survival and survival of higher risk patients would improve with the new strategy. Our second hypothesis was that the outcome of patients with lower risk relapse would not be worsened, even if many of them did not receive HSCT.

## Patients and Methods

### Patients

We reviewed the records of all patients with refractory or first relapse ALL aged from 1 to 18 years at initial diagnosis, having begun their first-line chemotherapy at our institution (Sainte Justine University Health Center, SJUHC) between January 1996 and December 2011, and having relapsed until December 2012. Patients were identified in the registry for leukemia of SJUHC. Data were extracted from each patient’s medical charts. Exclusions criteria were a diagnosis of mature B-cell leukemia, ALL associated with a genetic background (i.e. Down syndrome, ataxia-telangiectasia), secondary ALL. The study was approved by the Institutional Review Board (Research Ethics Board of Sainte-Justine University Hospital Research Center). The approval IRB number was 3765. As this study was retrospective, an informed consent was not obtained. Patient informations were anonymized and de-identified prior to analysis.

Patients had initially been treated according to DFCI consortium protocols 95, 00 or 05, usually for a total of 24 months. Those 3 protocols contain an induction-course therapy whose cytotoxic component was an identical combination of vincristine, doxorubicin, L-asparaginase, methotrexate, and glucocorticoids [12–14]. Dexrazoxane was given to a randomized half of patients on DFCI 95–01 and given to all patients on DFCI 2000–01. Induction was followed by an intensification course containing the same drugs as well as 6-mercatopurine, and then continuation therapy until 2 years after complete remission (CR). Central nervous system (CNS) prophylaxis included intrathecal chemotherapy with or without cranial irradiation, depending on the risk and year of protocol. Since 2003, patients with Philadelphia chromosome positive leukemia also received a tyrosine kinase inhibitor.

### Definitions and response criteria

Patients were considered to have achieved CR if treatment resulted in a M1 marrow (≤5% blasts by bone marrow aspirate), without evidence of circulating blasts or extramedullary disease and with recovery of peripheral counts. Patients were classified as refractory when marrow still had more than 5% of lymphoblasts at the end of induction. Relapse was defined as a confirmed M2 or M3 marrow (≥5% leukemic blasts) or the presence of leukemia in any other site (eg, CNS, testis) in a patient who previously had achieved CR. Isolated extramedullary relapse was diagnosed on a clinically-overt extramedullary manifestation of leukemia and less than 5% marrow infiltration. CNS relapse was diagnosed in case of at least five leukocytes per microliter CSF and presence of lymphoblasts. Testicular relapse was confirmed by biopsy. A combined relapse was defined as the association of an extramedullary and a marrow relapse. For transplanted patients, treatment-related mortality was defined by death without leukemia recurrence.

**Fig 1:**
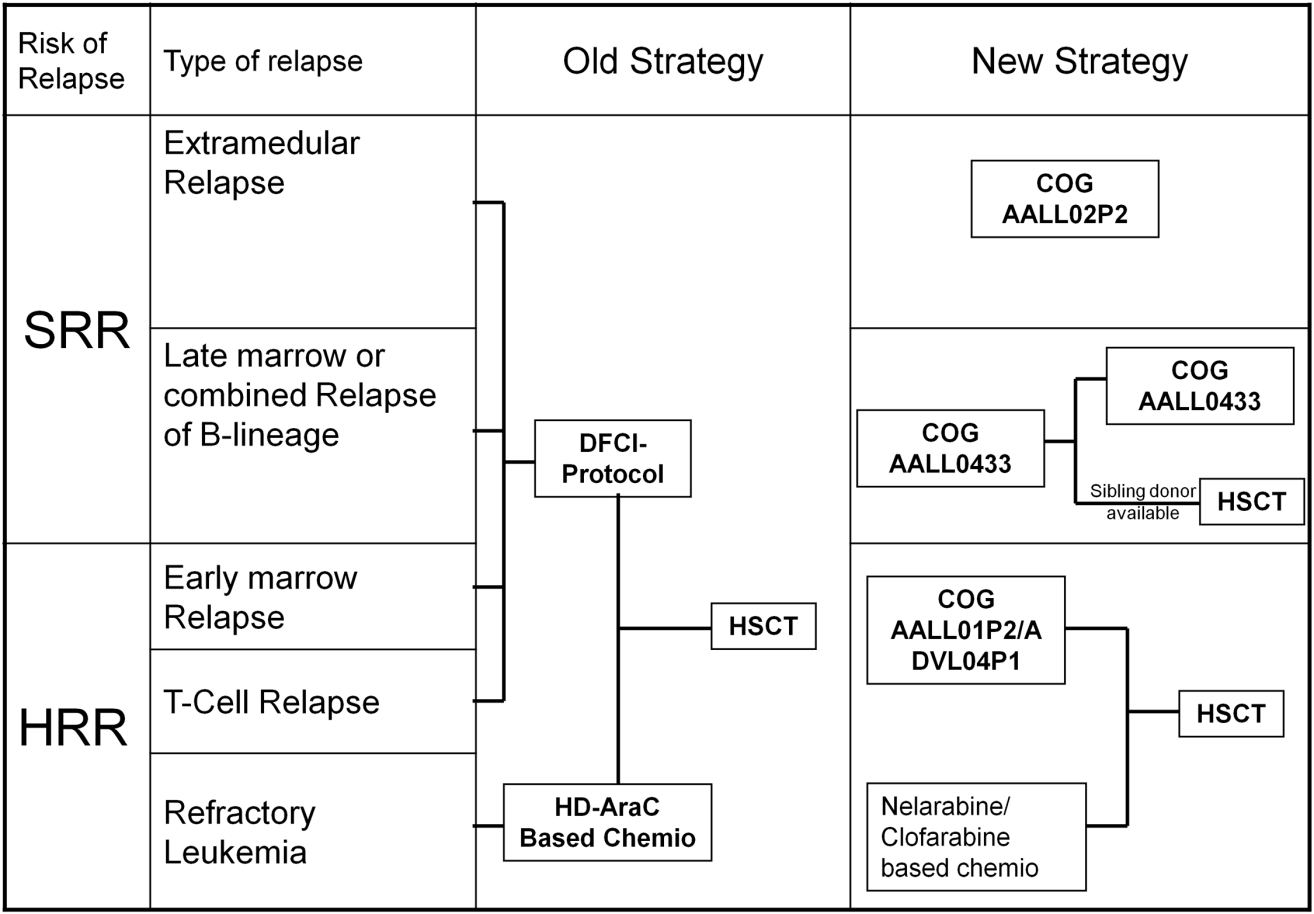
Previous and new treatment strategies for standard risk relapse (SRR) and High risk relapse or refractoriness (HRRR), before and after 2007. HSCT, haematopoietic stem cell transplantation; AraC, Cytarabine; COG, Children Oncology Group; DFCI, Dana-Farber Cancer Institute.

Patients were classified into two groups (Fig 1). Isolated extramedullary relapse of any lineage, marrow or combined relapse of B-lineage ALL occurring more than 36 months after initial diagnosis were classified as standard risk relapses (SRR). T-lineage, marrow or combined relapse of B-lineage ALL occurring within 36 months from diagnosis, refractory ALL (any lineage) were classified as high-risk relapse or refractory disease (HRRR).

### Treatment

Two different treatment strategies have been used (Fig 1). Before 2007, all the relapsing patients were uniformly treated with reinduction chemotherapy according to the current Dana Farber Cancer Institute (DFCI) protocol (similar to the one used at presentation). For patients with refractory leukemia a reinduction with high-dose cytarabine-based chemotherapy was used. HSCT with a related or unrelated donor without further chemotherapy (Fig 1), irrespective of their risk of second relapse, was offered to all fit patients reaching CR after reinduction. There was no predefined strategy for patients without CR after reinduction.

After 2007, SRR patients were treated with chemotherapy alone, except for pre-B late marrow relapse patients with a HLA-identical sibling (Fig 1). COG (Children’s Oncology group) AALL0433 was used for pre-B late marrow and early extra-medullary relapses. AALL02P2 or POG9412 was used for late extra-medullary relapses. HRRR patients received at least 3 cycles of chemotherapy. These 3 cycles of re-induction chemotherapy were based on the COG protocol AALL01P2 [5] or ADVL04P1 (with Epratuzumab). Patients in CR after these 3 cycles proceeded immediately to HSCT. For relapse with T-lineage leukemia or refractory pre-B leukemia nelarabine- or clofarabine-based regimen were used, respectively. There was no predefined strategy for patients without CR after the 3 cycles of chemotherapy.

HSCT donor search policy (priority for cord blood over bone marrow for unrelated donors), conditioning regimen (12Gy fractionated-TBI associated with cyclophosphamide and etoposide or fludarabine), serotherapy before HSCT (ATG for unrelated HSCT) and graft-versus-host disease (GvHD) prophylaxis (cyclosporin A associated with methotrexate for bone marrow/PBSC or steroids for cord blood) remained unchanged for patients treated before and after 2007.

### End points and statistical methods

Analysis was performed using March 31, 2014 as the cut off date for follow-up. No patients were lost to the follow-up. Median and range (minimum/maximum) or frequency and percentage were collected for all study variables. Differences between variables were analyzed by Chi-square/Fisher exact test for categorical variables.

Event-free survival (EFS) was the primary endpoint. EFS was calculated as the interval between the date of the first relapse/refractory and the date of the first event. An event was defined as any of the following: absence of CR after reinduction, second relapse, second malignancy, or death from any cause. Overall survival (OS) was defined as the time from diagnosis of the first relapse or refractory disease to the death due to any cause. Probability of EFS and OS were calculated according to the Kaplan-Meier’s method and compared by the log-rank test.

Variables with a p<0.10 in univariate analysis (Gender, WBC, Genetics risk group, Risk group of relapse, Treatment strategy) were included in a multivariate Cox regression model alongside with relapse date (before or since 2007). Statistical analyses were performed with SPSS version (IBM Inc, Chicago, IL) software package. A two-tailed P < 0.05 was considered as statistically significant.

## Results

Between 1996 and 2011, 453 children were diagnosed with ALL at our institution. All were treated with one of the above-mentioned DFCI protocols. Of the 453 patients, 64 were subsequently diagnosed with a first relapse (n = 55) or refractory (n = 11) ALL before December 2012. At initial presentation, median age was 6.7 (range 1.3–17.2) years and median WBC count was 20 (1.9–599) x10^9^/L. Characteristics of the 64 patients at first presentation are shown in the Table 1.

**Table 1:** Characteristics at first presentation of ALL patients who subsequently relapsed or were refractory to first-line induction.

*Characteristics*	*No*. *of Patients (n = 64)*	%
**Age, years**		
1–9	47	73
≥ 10	17	27
**WBC counts/μL**		
< 50.000	48	75
≥ 50.000	16	25
**Gender**		
Female	21	33
Male	43	67
**Risk criteria**		
Standard risk	26	41
High risk	30	47
Very high risk*	8	12
**Mediastinal Mass**		
Yes	6	9
No	58	91
**CNS disease**		
Yes	21	33
No	43	67
**Immunophenotype**		
Pre B cell	54	84
T cell	10	16
**Genetics**		
Normal and unknown	30	47
Without prognosis	10	16
Bad prognosis^+^	8	12
Good prognosis^#^	16	25

*Cytogenetic of bad prognosis and positive MRD at the end of induction,

^+^Hypodiploid (≤<44 chromosomes), MLL rearrangements, BCR-ABL1,

^#^ETV6-RUNX1 and Hyperdiploid (>50 chromosomes)

Characteristics of patients at diagnosis of relapse/refractory disease are presented in the Table 2. Median age at diagnosis of relapse or refractory disease was 9.3 (range 1.3–18.3) years. Relapsing patients were younger than patients with refractory disease: median age 5.9 versus 8.4 years, respectively. Regarding only relapsed patients, median WBC blood count was 6 x 10^9^/L and there were 30 (56%) isolated marrow, 12 (23%) combined and 11 (21%) extramedullary relapses.

**Table 2:** Characteristics of relapsed or refractory patients at diagnosis of relapse or refractory disease.

Characteristics	Before 2007	After 2007	Total	p
	n (%)	n (%)	n (%)	
	39	25	64	
**Risk at presentation:**				
Standard	15 (39)	11 (44)	26 (41)	0.6
High	20 (51)	10 (40)	30 (47)	
Very high risk	4 (10)	4 (16)	8 (12)	
**Immunophenotype:**				
B	33 (85)	21 (84)	54 (84)	0.9
T	6 (15)	4 (16)	10 (16)	
**Type of relapse/refractory disease:**				
Medullary	24 (62)	15 (60)	39 (61)	0.9
Extramedullary	6 (15)	5 (20)	11 (17)	
Combined	9 (23)	5 (20)	14 (22)	
**Risk at relapse:**				
SRR	21 (54)	9 (36)	30 (47)	0.2
HRRR	18 (46)	16 (64)	34 (53)	
**HSCT:**				
Yes	32 (82)	16 (64)	48 (75)	0.1
No	7 (18)	9 (36)	16 (25)	
**Cytogenetics:**				
Good prognosis	10 (26)	6 (24)	16 (25)	0.8
Normal-Unknown-Without prognosis	25 (64)	15 (60)	40 (63)	
Bad Prognosis	4 (10)	4 (16)	8 (12)	

SRR, standard risk relapse; HRRR, high risk relapse or refractoriness.

The median duration of first remission for the whole group was 32.5 (0.6–135) months. 30 (47%) were classified as SRR and 34 (53%) as HRRR, including eleven (17%) with refractory ALL. As shown in the Table 2, there was no significant difference between patients who relapsed before 2007, treated with the previous uniform approach, and patients who relapsed since 2007, treated with the new risk-based strategy.

Of the 64 patients, 48 received an allogeneic HSCT, most of them (40 patients) after a total body irradiation-based conditioning regimen. The main source of hematopoietic stem cells was cord blood (CB—n = 31, all unrelated, except one). Before 2007, 32 (82%) patients received an allogeneic HSCT. Cause of not receive a HSCT before 2007 (n = 7) were refractory disease (n = 2), co-morbidities (n = 2), medical decision (n = 2) and death during induction (n = 1). After 2007, 16 (64%) patients received an allo-HSCT. The median follow-up after the diagnosis of first relapse or refractory disease was 28 (1–152) months for the whole population and 61 (16–152) months for the patients alive.

Fifty-nine (92%) patients achieved second remission. During reinduction, four patients (6%) died of progressive disease and one from a toxic death. Overall, 35 patients died, 25 (39%) from disease progression and 10 (16%) related to treatment. The relapse rate of transplanted patients decreased from 41% (before 2007) to 31% (after 2007), although HSCT was offered only to high-risk relapse (HRRR) patients since 2007. Post-transplant relapse rate of these HRRR patients decreased from 56% (before 2007) to 31% (after 2007) (P = 0.02). Transplantation-related mortality also decreased from 25% (before 2007) to 6% (after 2007—P = 0.1).

**Fig 2:**
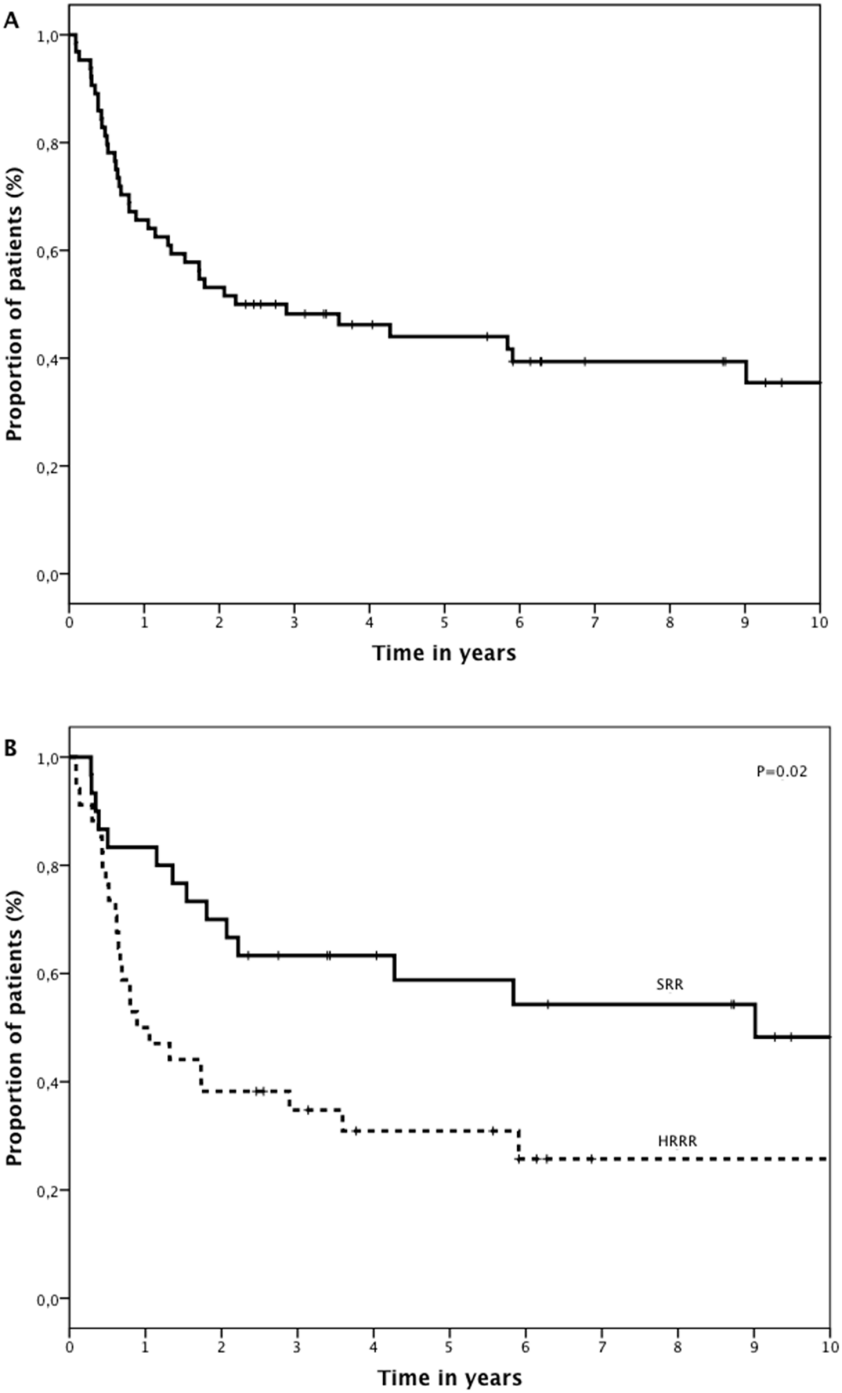
Survival of all relapsed and refractory ALL patients. (A) 5- years EFS for all patients: 44% (B) 5 years EFS for standard risk relapse (SRR): 59% versus high risk relapse or refractory (HRRR): 31%.

**Fig 3:**
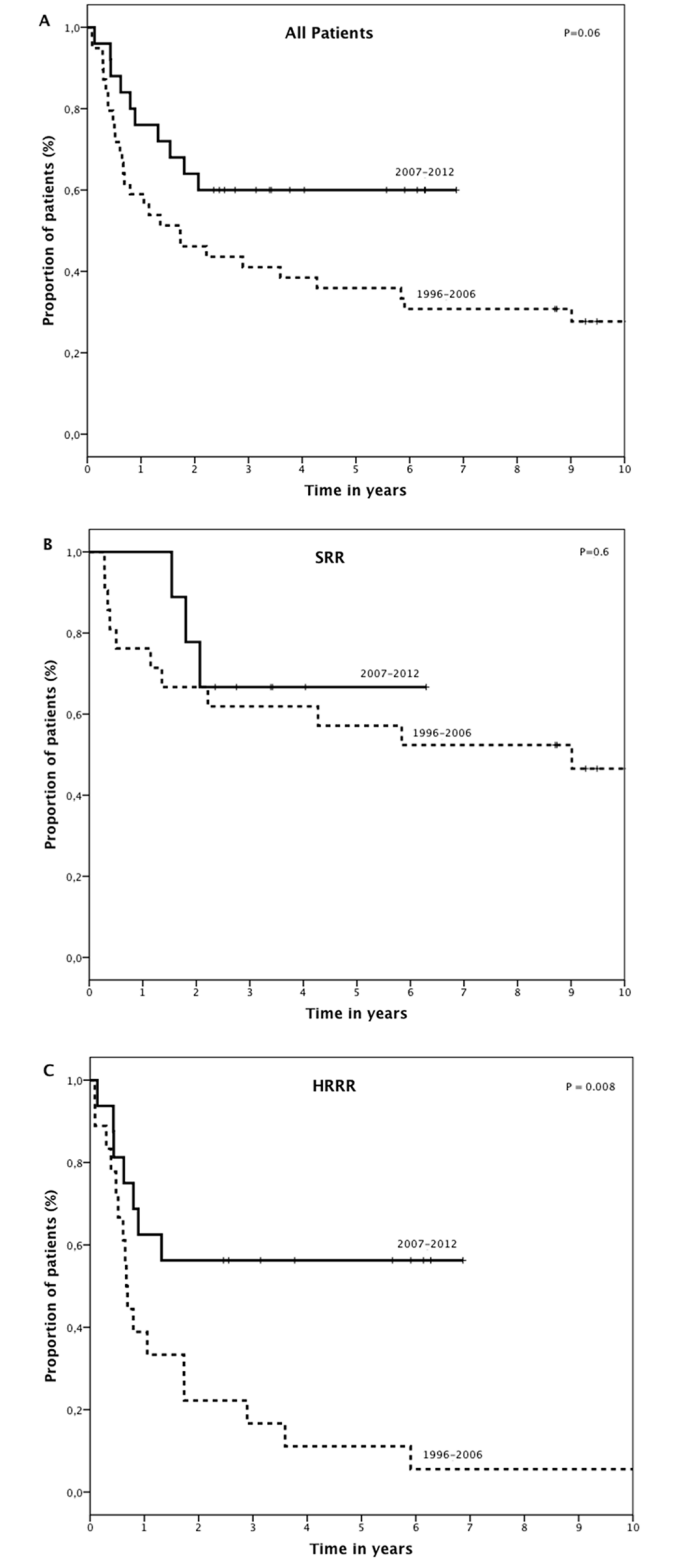
EFS by strategy. Before 2007, all relapsed patients were offered an hematopoietic stem cell transplantation (HSCT) after a single cycle of chemotherapy. After 2007, HSCT was offered to patients with high risk relapse, after at least 3 cycle of chemotherapy (A) 5-year EFS of all relapses or refractoriness: 41% vs 60%, (B) 5-years EFS of standard risk relapses (SRR): 57% vs 67%, and (C) 5-years EFS of high risk relapses or refractory (HRRR): 11% vs 56%.

Five-year estimated EFS for the whole population was 44% (Fig 2). Five-year EFS was 59% for SRR and 31% for HRRR (P = 0.02; Fig 2). Five-year EFS was 36% and 60% before and after 2007, respectively (p = 0.06; Fig 3). There was no difference in EFS before and after 2007 for SRR (57% vs 67%, respectively, p = 0.6; Fig 3). For HRRR, 5-year EFS was 11% and 56% before and after 2007, respectively (p = 0.008; Fig 3). Gender (p = 0.002), cytogenetic risk group (p = 0.02), date of relapse (before vs. after 2007—p = 0.03) and risk group (p = 0.03), were associated with EFS (Table 3) in multivariate analysis.

**Table 3:** Multivariate analysis of survival and event free survival for relapsed or refractory ALL.

	Overall Survival			Event-free survival		
Variable	P	Hazard Ratio (HR)	CI 95% for HR	P	Hazard Ratio (HR)	CI 95% for HR
**Gender (female)**	0.04	2.22	1.04–4.74	0.02	2.34	1.11–4.91
**WBC (≥50x10**^**9**^**/L)**	0.5	1.37	0.59–3.14	0.06	2.14	0.96–4.79
**Genetics risk group**	0.2	0.83	0.61–1.13	0.23	0.84	0.63–1.12
**Risk group at relapse (high risk relapse / refractory)**	0.008	0.33	0.15–0.76	0.05	0.49	0.23–1.01
**Treatment strategy (risk based strategy [2007–2012])**	0.05	0.43	0.19–0.99	0.12	0.54	0.25–1.17

The referent categories were Gender (Male vs. Female), WBC (<50 vs. ≥50x10^9^/L at initial presentation), Genetic risk group (Normal / unknown vs. without prognosis vs. Bad prognosis vs. Good prognosis), risk group at relapse (standard risk relapse vs. high risk relapse / refractory), treatment strategy (uniform approach (1996–2006) vs. risk based strategy [2007–2012]).

Estimated five-year overall survival for the whole group was 47%. Five-year survival was 63% for SRR and 33% for HRRR (p = 0.006). Five-year survival was 41% and 59% before and after 2007, respectively (p = 0.13). There was no difference in survival before and after 2007 for patients with SRR (P = 0.7). However, 5-year survival was significantly higher after 2007 for HRRR: 55% versus 17% (p = 0.03). In multivariate Cox regression model, date of relapse (before or after 2007) was significantly associated with survival (p = 0.03). Gender and risk group were also associated with overall survival (p = 0.02 and p = 0.008, respectively, Table 3).

## Discussion

We compared two different strategies to treat relapsed or refractory ALL. The previous strategy was uniform: all patients underwent HSCT after a single cycle of chemotherapy. The new strategy took into account the risk of second relapse: most SRR patients were treated with chemotherapy alone, whereas HRRR patients underwent HSCT after 3 cycles of chemotherapy. Overall survival and EFS of SRR patients were identical. However, OS and EFS of HRRR patients were significantly better with the risk-based strategy, and became similar to those of SRR patients. The change for a risk-based strategy was motivated by several factors. First, the result of our previous uniform strategy was particularly dismal in HRRR patients, with an estimated 5-years survival of 13% for patients with early marrow relapse [10]. Second, other institutions had already shown that, in standard risk patients, chemotherapy alone can achieve results comparable to those of HSCT [15]. A further advantage of avoiding HSCT is to spare HSCT-related mortality and long-term complications, particularly those related to total-body irradiation or chronic GvHD. Third, patients with higher levels of MRD before HSCT are more likely to relapse after HSCT [11, 16, 17]. Fourth, early MRD response at the end of reinduction treatment is a strong predictor of outcome [9, 18], and MRD shows a continued regression with subsequent blocks of therapy in a significant number of patients [5]. Fifth, *in vitro* data suggest resistance of leukemic cells at relapse to standard chemotherapy agents [19, 20]. Taken together, these data prompted us to treat standard risk patients with chemotherapy alone, and to intensify pre-transplant chemotherapy for higher risk patients, exposing them to drugs not given at presentation.

The improvement of the outcome we observed in higher risk patients can be largely attributed to both the decrease in post-transplant relapse rate and a reduction in transplant-related mortality. Of note, most of our HSCT protocols have remained unchanged between the two periods, including the rate of cord blood to bone marrow grafts, conditioning regimen, and GvHD prophylaxis. Although pre-transplant MRD was not measured in most of these patients, we believe that the main factor influencing a lower relapse rate is the more intensive pre-transplant chemotherapy. The improvement in transplant-related mortality can rather be attributed to the general improvement in the supportive care, particularly in infectious disease monitoring and treatment, such as widespread access to viral detection techniques (i.e. access to new tests, such as PCR [qualitative or quantitative] as well as increase in the frequency of testing) and easiest access to antiviral agents (foscarnet, cidofovir) [21]. Furthermore, access to defibrotide for venooclusive disease was also improved by having a start-up treatment stock in place.

This study has two main limitations. First, it is limited to a single institution. However, our rates of relapse, survival and EFS are in the range of those published by other institutions and multicentric trials [4–7, 15, 22–25]. In particular, five-year EFS/OS were 59% and 63% for SRR in our cohort and these results that are very similar to COG AALL0433 trial, where the 3-year EFS/OS for the entire cohort (271 patients) were 61.4 ± 4.3% and 72.9 ± 3.9% respectively [26].

Second, the two strategies have been used successively. Some of the differences reported here can thus be attributed to a historical improvement in other factors, particularly in supportive care after HSCT [21]. Unfortunately, specific post-HSCT outcomes/causes of death such as GVHD, VOD, infections are not available for comparison of the two eras to better illustrate this point. Nevertheless, our study demonstrated that, in the context of the general evolution of the field during the 1996–2012 period, changing for a risk-based strategy in the treatment of relapsed or refractory ALL leads to an improvement of higher risk patient outcome without jeopardizing the outcome of standard risk patients.

Despite the improvements obtained by taking risk into account when it comes to devise treatment for relapsed ALL, outcomes of relapsed and refractory ALL is still an issue [5–7]. Current studies have already taking into account reduction of pre-transplant disease burden, taking advantage of the larger availability of MRD measurement techniques. Further improvement can also rely on new approaches, such as post-transplant targeted therapy or immunotherapy [1, 27–33].
